# Palliative Care Specialist Use Among Medicare Decedents Who Had Poor-Prognosis Cancers

**DOI:** 10.1001/jamanetworkopen.2025.22886

**Published:** 2025-07-24

**Authors:** Isaac S. Chua, Haiden A. Huskamp, Ateev Mehrotra, Andrew D. Wilcock

**Affiliations:** 1 Division of General Internal Medicine and Primary Care, Department of Medicine, Brigham and Women’s Hospital, Boston, Massachusetts; 2 Department of Psychosocial Oncology and Palliative Care, Dana-Farber Cancer Institute, Boston, Massachusetts; 3 Harvard Medical School, Boston, Massachusetts; 4 Department of Health Care Policy, Harvard Medical School, Boston, Massachusetts; 5 Department of Health Services, Policy, and Practice, Brown School of Public Health, Providence, Rhode Island

## Abstract

**Importance:**

Palliative care (PC) use patterns may have changed in recent years due to increased adoption of telehealth and the availability of more advanced practice clinicians who specialize in PC delivery.

**Objective:**

To describe changes in the use of specialty PC during the last year of life among Medicare beneficiaries who had cancers with poor prognoses (cancers that commonly caused death, rare cancers with high mortality rates, or solid tumors with concurrent nonlymphatic metastases; hereinafter termed *poor-prognosis cancers*).

**Design, Setting, and Participants:**

This retrospective cohort study includes all US Medicare fee-for-service beneficiaries who died from poor-prognosis cancers between January 1, 2018, and December 31, 2023, and received care in hospital and outpatient settings.

**Exposures:**

Encounters with a PC specialist.

**Main Outcomes and Measures:**

The primary outcome was the proportion of decedents with any specialty PC encounter in their last year of life. Secondary outcomes included mean number of PC encounters among decedents with at least 1 encounter with a PC specialist and telehealth use. PC specialists were clinicians who self-identified as a PC specialist or were clinicians with 80% or more of their Medicare encounters focused on PC.

**Results:**

The cohort included 1 508 103 decedents (mean [SD] age, 79.6 [8.0] years; 54.6% male) with poor-prognosis cancers. Between 2018 and 2023, the proportion of decedents with at least 1 PC encounter increased from 29.84% to 37.21% (adjusted change, 7.21 [95% CI, 6.30-8.12] percentage points; relative change, 24.2%). The proportion who received outpatient PC increased from 10.66% to 20.56% (adjusted change, 9.41 [95% CI, 8.33-10.48] percentage points; relative change, 88.2%). In 2023, 22.84% of all decedents received PC from advanced practice clinicians vs 15.60% by self-designated PC physicians and 9.92% by other physicians. Telehealth was used for 18.2% of all outpatient palliative care encounters in 2023. Decedent characteristics associated with not receiving specialty PC included older age, lower income, and living in nonmetropolitan areas.

**Conclusions and Relevance:**

In this cohort study of decedents who had poor-prognosis cancers, an increasing proportion received any specialty PC. Advanced practice specialists were the most common clinician type who delivered specialty PC, and telehealth was used for a substantial proportion of outpatient visits. Despite these changes, only a minority of patients received specialty PC, and low use of specialty PC among certain subpopulations persisted, suggesting that different strategies are needed to overcome these barriers.

## Key Points

**Question:**

Has specialty palliative care (PC) use among Medicare decedents who had cancers with poor prognoses changed in the context of greater telehealth use and more advanced practice clinicians in the field?

**Findings:**

In this cohort study of 1 508 103 Medicare decedents, the proportion with specialty PC use increased 24% from 2018 to 2023, largely driven by outpatient encounters and care by advanced practice clinicians. Decedents who were older, had lower incomes, and were living in nonmetropolitan areas remained less likely to receive any PC.

**Meaning:**

These findings suggest that different strategies are needed to increase PC use among some disadvantaged subpopulations.

## Teaser

This cohort study describes changes in the use of palliative care specialists among patients with poor-prognosis cancers in their last year of life among all deceased traditional Medicare beneficiaries in the US between 2018 and 2023.

## Introduction

Early palliative care (PC) is considered the standard of care for patients with advanced cancer and is associated with improved quality of life and symptom control.^1,2,3^ The American Society of Clinical Oncology recommends that oncologists refer patients with advanced cancer to specialized PC early in the disease course.^2^ Unfortunately, as of 2016, only a minority of patients with advanced cancer received PC.^4^

Recent changes may have increased PC use. First, outpatient PC is increasingly recognized as a key component for delivering early, concurrent PC services for patients with advanced cancer receiving cancer-directed therapies.^2,5,6,7,8^ Second, the COVID-19 pandemic changed patterns of health care use,^9,10^ catalyzing the rapid adoption of telehealth,^11,12,13,14,15^ which has been shown to be as effective as in-person PC for patients with advanced lung cancer.^16^ Third, advanced practice clinicians (APCs), such as nurse practitioners and physician assistants, are playing a larger role in the health care workforce overall.^17,18^ Within PC, APCs may be providing more PC services to alleviate the shortage of specialty PC physicians.^19,20^

Given this context, we describe changes in PC specialist use among patients with cancer with poor prognoses (hereinafter termed *poor-prognosis cancers*) in their last year of life among all deceased traditional Medicare beneficiaries in the US between 2018 and 2023. Specifically, we described changes by care modality (in person vs telehealth), setting (hospital vs outpatient), and PC clinician type. We also explored the association between decedent characteristics and receipt of PC and how these associations have changed over time.

## Methods

### Data Source

This cohort study used 100% fee-for-service Medicare inpatient, outpatient, and professional claims data from January 1, 2018, to December 31, 2023, to analyze in-person and telehealth use of PC specialists in the final 12 months of life among traditional Medicare beneficiaries who died from poor-prognosis cancers. The Harvard Medical School Institutional Review Board approved this study and waived the need for informed consent for the use of secondary data. We followed the Strengthening the Reporting of Observational Studies in Epidemiology (STROBE) reporting guideline.

### PC Specialists

Our focus was on nonhospice PC encounters delivered by clinicians who focus on hospice and palliative medicine.^4,21,22^ They included specialty-trained PC physicians or other clinicians who may or may not have gone through PC training but who function as PC specialists by way of their practice patterns and clinical focus.^23^ Specifically, we defined PC clinicians as any clinician (identified with a unique National Provider Identification number) who met at least 1 of the following 2 criteria: (1) self-designation using Centers for Medicare & Medicaid Services (CMS) specialty code 17 for hospice and palliative care on their Medicare enrollment application (hereafter referred to as self-designated PC physicians), or (2) at least 80% of their evaluation and management (E&M) encounters during a given year (among those with a minimum of 30 E&M encounters) had an *International Statistical Classification of Diseases and Related Health Problems, Tenth Revision* (*ICD-10*), code Z51.5 for encounter with PC. E&M encounters were identified in the carrier claims using the Berenson-Eggers Type of Service classification of the Healthcare Common Procedure Coding System codes for E&M care.^24^ Multiple claims per patient-clinician-day were counted as 1 encounter.

Using CMS specialty codes to identify specialty-trained physicians (including PC physicians) has good agreement (κ = 0.80) with the American Medical Association Physician Masterfile.^25^ Using the *ICD-10* code Z51.5 allowed us to identify other clinicians whose clinical practice focuses on delivering PC services. There is no CMS specialty code for APCs who specialize in PC. Rather, almost all APCs are given generic designations of nurse practitioner and physician assistant (codes 50 and 97, respectively). There are also physicians who do not self-identify as PC specialists on their Medicare enrollment application (National Provider Identification numbers with any other CMS specialty code) but clinically focus on PC.

Consistent with prior work categorizing APCs into specialties,^26^ we set a high cutoff of at least 80% of E&M encounters during a given year to minimize misclassifying non-PC specialists who may occasionally use this billing code for PC-related services that a nonspecialist could also perform (eg, transitioning a patient to comfort measures).^27,28^ If a clinician met the criteria for being a PC specialist in any year, we considered them a specialist in every year of our study. eMethods 1 in Supplement 1 provides more details on identifying PC specialists.

### Study Population

We identified decedents 66 years or older continuously enrolled in fee-for-service Medicare Parts A and B for at least 12 months prior to their death and who likely died from causes related to a poor-prognosis cancer. Our definition of poor-prognosis cancers is consistent with prior studies that included the 10 most common causes of cancer death per the American Cancer Society^29^ and National Vital Statistics System^30^ plus rare cancers with high mortality rates (eMethods 2 in Supplement 1).^31,32^ Solid tumors commonly diagnosed at early stages (eg, breast, prostate, and colorectal) were required to have a concurrent nonlymphatic metastatic disease code to be considered a poor-prognosis cancer.^31,32^ To identify individuals who likely died of cancer instead of individuals who died from another cause but also had a history of cancer, each cancer was identified by searching the decedents’ *ICD-10* diagnosis codes on their inpatient, outpatient, and professional claims from the prior 2 years with at least 1 inpatient or at least 2 outpatient or professional encounters that met the previously described criteria for a poor-prognosis cancer.

Using the Master Beneficiary Summary File, we obtained information on the decedents’ age, sex, race and ethnicity, rural-urban categorization of their zip code of residence (ie, rural-urban commuting area codes 1 to 3 were considered metropolitan and the remaining codes were considered nonmetropolitan),^33^ original reason for Medicare entitlement (eg, age, disability, or end-stage kidney disease), Medicaid enrollment status, US Census region of residence, and presence of 30 chronic condition indicators as defined by the Chronic Conditions Data Warehouse. Race and ethnicity were categorized from the Medicare enrollment file as self-reported Asian, Black, Hispanic, White, and other (including American Indian or Alaska Native, Native Hawaiian or Pacific Islander, and unknown) and included given their historical association with differences in health care outcomes and access to health care services. Dual enrollment with Medicaid was used as a marker of low income.^34^

### Settings

Settings were identified using the restructured Berenson-Eggers Type of Service classification subcategories for E&M Healthcare Common Procedure Coding System codes on the Carrier claim. Hospital setting included claims with subcategories for hospital inpatient services, emergency department services, observation care services, and critical care services; outpatient setting included subcategories for hospice or palliation services, office or outpatient services, home services, care management or coordination services, behavioral health services, ophthalmological services, and miscellaneous services. Claims for nursing facility services were rare and did not fit under the outpatient or hospital setting definitions and were omitted as another setting we evaluated.

### Outcomes

Outcomes were identified during a 1-year period before a patient’s death. Our main outcome was a dichotomous indicator for receipt of 1 or more E&M encounters with a PC specialist in the decedent’s last year of life. We considered any E&M encounter with a PC specialist as a PC specialist encounter, whether a Z51.5 diagnosis code was used or not during the encounter. Secondary outcomes included the count of E&M encounters with a PC specialist in the last year of life. At the encounter level, we categorized whether the PC encounter occurred in a hospital vs an outpatient setting; whether it was delivered by a self-identified PC physician, APC, or other physician; and whether it was delivered via telehealth. We identified telehealth use by place of service codes and Healthcare Common Procedure Coding System codes and modifiers for audio-only or video telehealth encounters (eMethods 3 in Supplement 1).

We focused on the care received by decedents prior to any hospice enrollment. While hospice and PC are considered part of the same specialty, hospice services are traditionally not delivered early in a patient's disease trajectory while they are receiving active cancer treatment,^35^ and hospice use is often assessed as an outcome separate from PC specialist encounters.^36^

### Statistical Analysis

Our analytic data were at the decedent level, organized by date of death. Our analysis proceeded in 3 parts. First, we plotted unadjusted patterns in our outcome means according to decedent month and year of death. Second, to summarize changes in our outcomes during our 5-year study period, we estimated the adjusted difference in our outcomes between the first and last years of our study period using regression models that included decedents from years 2018 and 2023 only. These models included an indicator for death in 2023 and controlled for decedent characteristics (including cancer type, age, sex, race and ethnicity, Medicaid enrollment status, reason for Medicare enrollment, rurality, US Census region, and number of comorbidities) with clustered SEs by decedent state of residence. We used linear regression for our continuous outcomes and logistic regression, reporting marginal effects, for our dichotomous outcomes.^37^ Third, we quantified what patient characteristics were associated with receipt of any PC in 2018 and 2023, and whether there was a change in these associations between the 2 years. For example, we quantified whether PC use between younger and older patients attenuated or grew further apart in 2023 compared with the difference in 2018. To do so, we estimated another logistic regression of having at least 1 vs 0 encounters with a PC specialist in years 2018 and 2023, including the same set of decedent characteristics described previously and an indicator for death in 2023; however, for this regression, we added interactions between each patient characteristic and death in 2023. Using the estimated coefficients from this model, we were able to assess changes by decedent characteristics in our outcome between those years.^37^

We used Stata, version 18.0 (StataCorp LLC) for all analyses. Significance was determined by 2-sided *P* < .05.

## Results

### Decedent Characteristics

The cohort included 1 508 103 decedents who had poor-prognosis cancers and died between 2018 and 2023 (eFigure in Supplement 1). The mean (SD) age of decedents was 79.6 (8.0) years; 684 930 (45.4%) were women and 823 173 (54.6%) were men. In terms of race and ethnicity, 31 286 (2.1%) were Asian, 109 177 (7.2%) were Black, 57 808 (3.8%) were Hispanic, 1 275 584 (84.6%) were White, and 34 248 (2.3%) were other race or ethnicity (Table 1). The most common cancer types were lung, gastrointestinal, genitourinary, other carcinoma (unknown), and breast. Most decedents lived in metropolitan settings (1 158 507 [76.8%]), and the largest proportion lived in the South (594 904 [39.4%]). The standardized differences in means between 2018 and 2023 for all decedent characteristics were all less than 0.1, indicating that the composition of the cohort changed little during the study period.

**Table 1.** Characteristics of Decedents Who Had Poor-Prognosis Cancers

Characteristic	No. (%)			Standardized differences in means between 2018 and 2023^a^
	All years (N = 1 508 103)	2018 (n = 260 714)	2023 (n = 229 746)	
Age, y				
Mean (SD)	79.6 (8.0)	79.4 (8.0)	79.8 (7.9)	0.05
66-69	197 696 (13.1)	36 338 (13.9)	27 418 (11.9)	0.06
70-74	301 898 (20.0)	52 337 (20.1)	44 300 (19.3)	0.02
75-79	314 806 (20.9)	53 639 (20.6)	49 523 (21.6)	0.02
80-84	289 715 (19.2)	49 042 (18.8)	46 207 (20.1)	0.03
85-89	227 023 (15.1)	39 634 (15.2)	34 833 (15.2)	0.001
≥90	176 965 (11.7)	29 724 (11.4)	27 465 (12.0)	0.02
Sex				
Female	684 930 (45.4)	119 483 (45.8)	104 804 (45.6)	0.004
Male	823 173 (54.6)	141 231 (54.2)	124 942 (54.4)	0.004
Race				
Asian	31 286 (2.1)	5016 (1.9)	5188 (2.3)	0.02
Black	109 177 (7.2)	20 661 (7.9)	14 386 (6.3)	0.07
Hispanic	57 808 (3.8)	10 089 (3.9)	8371 (3.6)	0.01
White	1 275 584 (84.6)	219 983 (84.4)	195 710 (85.2)	0.02
Other race^b^	34 248 (2.3)	4965 (1.9)	6091 (2.7)	0.05
Cancer type^c^				
Breast	149 690 (9.9)	24 813 (9.5)	24 091 (10.5)	0.03
Central nervous system	136 058 (9.0)	23 232 (8.9)	22 034 (9.6)	0.02
GI	496 249 (32.9)	83 962 (32.2)	77 691 (33.8)	0.03
Genitourinary	403 794 (26.8)	65 573 (25.2)	64 168 (27.9)	0.06
Leukemia	113 850 (7.5)	18 657 (7.2)	16 393 (7.1)	0.001
Lung	536 558 (35.6)	96 180 (36.9)	78 686 (34.2)	0.06
Melanoma	55 170 (3.7)	7922 (3.0)	9918 (4.3)	0.07
Ovarian and uterine	55 895 (3.7)	9301 (3.6)	8840 (3.8)	0.02
Other carcinoma	503 565 (33.4)	82 177 (31.5)	81 921 (35.7)	0.09
Geography				
Metropolitan	1 158 507 (76.8)	199 060 (76.4)	177 904 (77.4)	0.03
Nonmetropolitan	349 596 (23.2)	61 654 (23.6)	51 842 (22.6)	0.03
Medicaid eligibility				
Nondual	1 249 265 (82.8)	212 270 (81.4)	194 545 (84.7)	0.09
Dual	258 838 (17.2)	48 444 (18.6)	35 201 (15.3)	0.09
Original reason for entitlement				
Age	1 316 574 (87.3)	227 082 (87.1)	201 947 (87.9)	0.03
Disability	187 005 (12.4)	32 850 (12.6)	27 110 (11.8)	0.03
ESKD	4524 (0.3)	782 (0.3)	689 (0.3)	0.003
No. of chronic conditions^d^				
Mean (SD)	7.0 (3.6)	6.9 (3.5)	7.1 (3.6)	0.07
0-3	252 083 (16.7)	46 183 (17.7)	36 468 (15.9)	0.05
4-5	282 487 (18.7)	50 529 (19.4)	41 730 (18.2)	0.03
6-9	603 822 (40.0)	103 261 (39.6)	92 628 (40.3)	0.02
10-14	339 389 (22.5)	55 781 (21.4)	53 959 (23.5)	0.05
≥15	30 322 (2.0)	4960 (1.9)	4961 (2.2)	0.02
Census region				
Northeast	295 633 (19.6)	51 515 (19.8)	45 371 (19.7)	0.000
Midwest	343 914 (22.8)	60 183 (23.1)	50 750 (22.1)	0.024
South	594 904 (39.4)	103 608 (39.7)	89 584 (39.0)	0.02
West	273 652 (18.1)	45 408 (17.4)	44 041 (19.2)	0.05

Abbreviations: ESKD, end-stage kidney disease; GI, gastrointestinal.

^a^ Values larger than 0.1 standardized differences in means indicate a sizeable difference.

^b^ Includes American Indian or Alaska Native, Native Hawaiian or Pacific Islander, as well as unknown race; these are the options for self-identified race in the Medicare enrollment file besides White and Black race or Hispanic or Latino ethnicity. Less than 1% of beneficiaries are coded as unknown race.

^c^ Cancers were classified as either solid or liquid tumor cancers; a small percentage of decedents had concurrent solid and liquid tumor diagnoses.

^d^ For each decedent, we assessed the presence of 30 chronic conditions in the calendar year prior to death from the Chronic Conditions Data Warehouse.

### Changes in PC Specialist Use

**Figure 1.**
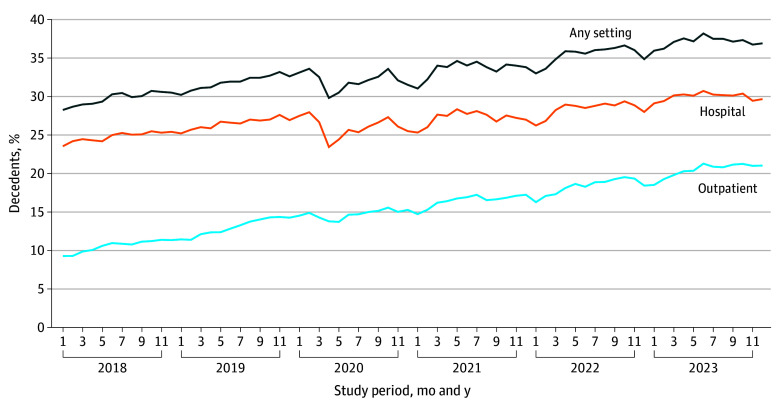
Patterns in the Share of Monthly Decedents Who Had Poor-Prognosis Cancers and at Least 1 Palliative Care Specialist Encounter Before Death by Care Setting The figure shows the share of decedents who had at least 1 palliative care specialist encounter in the year preceding their death date according to their month and year of death among decedents who had poor-prognosis cancers. If a decedent had both hospital and outpatient encounters, then they are only counted once in any setting.

From 2018 to 2023, the proportion of all decedents who received at least 1 specialty PC encounter in their last year of life increased from 29.84% to 37.21% (adjusted change, 7.21 [95% CI, 6.30-8.12] percentage points; relative change, 24.2%) (Table 2 and Figure 1). The proportion who received outpatient PC increased from 10.66% to 20.56% (adjusted change, 9.41 [95% CI, 8.33-10.48] percentage points; relative change, 88.2%), while the proportion who received hospital-based PC increased from 24.87% to 30.09% (adjusted change, 5.08 [95% CI, 4.25-5.90] percentage points; relative change, 20.4%).

**Table 2.** Changes in Encounters With Palliative Care Specialists Among Decedents Who Had Poor-Prognosis Cancers

Outcome	Decedents with ≥1 encounter				Encounters among decedents with ≥1 encounter			
	2018 (N = 260 714), %	2023 (N = 229 746), %	Adjusted change (95% CI), percentage points^a^	Relative change, %^b^	2018, Mean (SD) No.	2023, Mean (SD) No.	Adjusted change (95% CI), No.^a^	Relative change, %^b^
Palliative care encounter^c^	29.84	37.21	7.21 (6.30 to 8.12)	24.2	3.45 (3.66)	3.79 (4.12)	0.33 (0.26 to 0.40)	9.6
Setting^d^								
Outpatient	10.66	20.56	9.41 (8.33 to 10.48)	88.2	2.19 (2.28)	2.49 (2.51)	0.30 (0.21 to 0.39)	13.8
Hospital	24.87	30.09	5.08 (4.25 to 5.90)	20.4	3.19 (3.28)	3.36 (3.69)	0.17 (0.12 to 0.23)	5.4
Clinician type^e^								
Self-designated	14.06	15.60	1.25 (0.25 to 2.25)	8.9	3.06 (3.33)	3.25 (3.41)	0.15 (0.09 to 0.22)	5.0
Advanced practice clinicians	15.57	22.84	7.08 (6.19 to 7.97)	45.4	2.79 (2.72)	3.11 (3.23)	0.32 (0.24 to 0.40)	11.6
Other physicians	9.33	9.92	0.42 (−0.38 to 1.21)	4.5	2.80 (2.97)	2.93 (3.36)	0.1 (−0.03 to 0.22)	3.4

^a^ We modeled the outcome in 2018 and 2023 using a logistic regression for dichotomous outcomes and linear regression for continuous outcomes with a post indicator for 2023. Marginal effects of the post indicator are shown. Errors were clustered at the state level. Post estimates were adjusted by controlling for the characteristics shown in Table 1, including cancer type, age groups, sex, race and ethnicity, Medicaid enrollment status, reason for Medicare enrollment, rurality, Census region, and counts of comorbidities.

^b^ Relative changes are the adjusted change divided by the 2018 value.

^c^ Encounters are evaluation and management (E&M) services delivered by a clinician identified as a palliative care specialist in any study year.

^d^ The process for determining the setting appears in the Methods section.

^e^ The process for determining the clinician type is described in the Methods section.

Among decedents with at least 1 specialty PC encounter in the last year of life, the mean number of encounters per decedent increased from 3.45 to 3.79 (adjusted change, 0.33 [95% CI, 0.26-0.40]; relative change, 9.6%) (Table 2). Among those who received an encounter, the mean number of outpatient encounters per decedent increased from 2.19 to 2.49 (adjusted change, 0.30 [95% CI, 0.21-0.39]; relative change, 13.8%); and the mean number of hospital encounters increased from 3.19 to 3.36 (adjusted change, 0.17 [95% CI, 0.12-0.23]; relative change, 5.4%).

### Changes in How PC Was Provided

The proportion of all decedents who received PC from APCs increased from 15.57% to 22.84% (adjusted change, 7.08 [95% CI, 6.19-7.97] percentage points; relative change, 45.4%) (Table 2). In contrast, the proportion of all decedents who received PC from a self-designated PC physician increased from 14.06% to 15.60% (adjusted change, 1.25 [95% CI, 0.25-2.25] percentage points; relative change, 8.9%); the proportion who received PC from another type of physician increased from 9.33% to 9.92% (adjusted change, 0.42 [95% CI, −0.38 to 1.21] percentage points; relative change, 4.5%).

**Figure 2.**
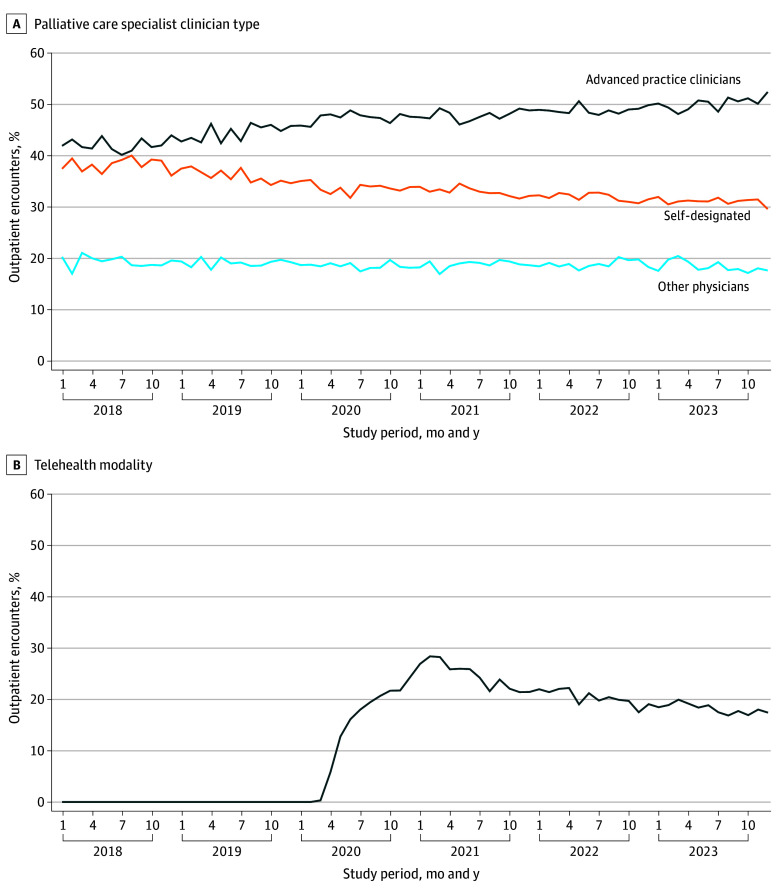
Patterns in the Share of Outpatient Encounters With a Palliative Care Specialist Among Decedents Who Had Poor-Prognosis Cancers The figure shows patterns in the share of outpatient specialist encounters according to the specialist's clinician type and the share of telehealth use during the year preceding the beneficiary's death date.

Between 2018 and 2023, APCs delivered a growing proportion of outpatient PC encounters, while the proportion of outpatient PC encounters delivered by self-designated PC physicians declined (Figure 2). The emergence of telehealth utilization for PC delivery began near the onset of the COVID-19 pandemic (March 2020) and increased until the beginning of 2021 (Figure 2). The proportion of PC visits provided by telehealth declined from a peak of 28.5% in February 2021, and by 2023 constituted 18.2% of all outpatient PC encounters.

### PC Specialty Use by Decedent Characteristics

**Figure 3.**
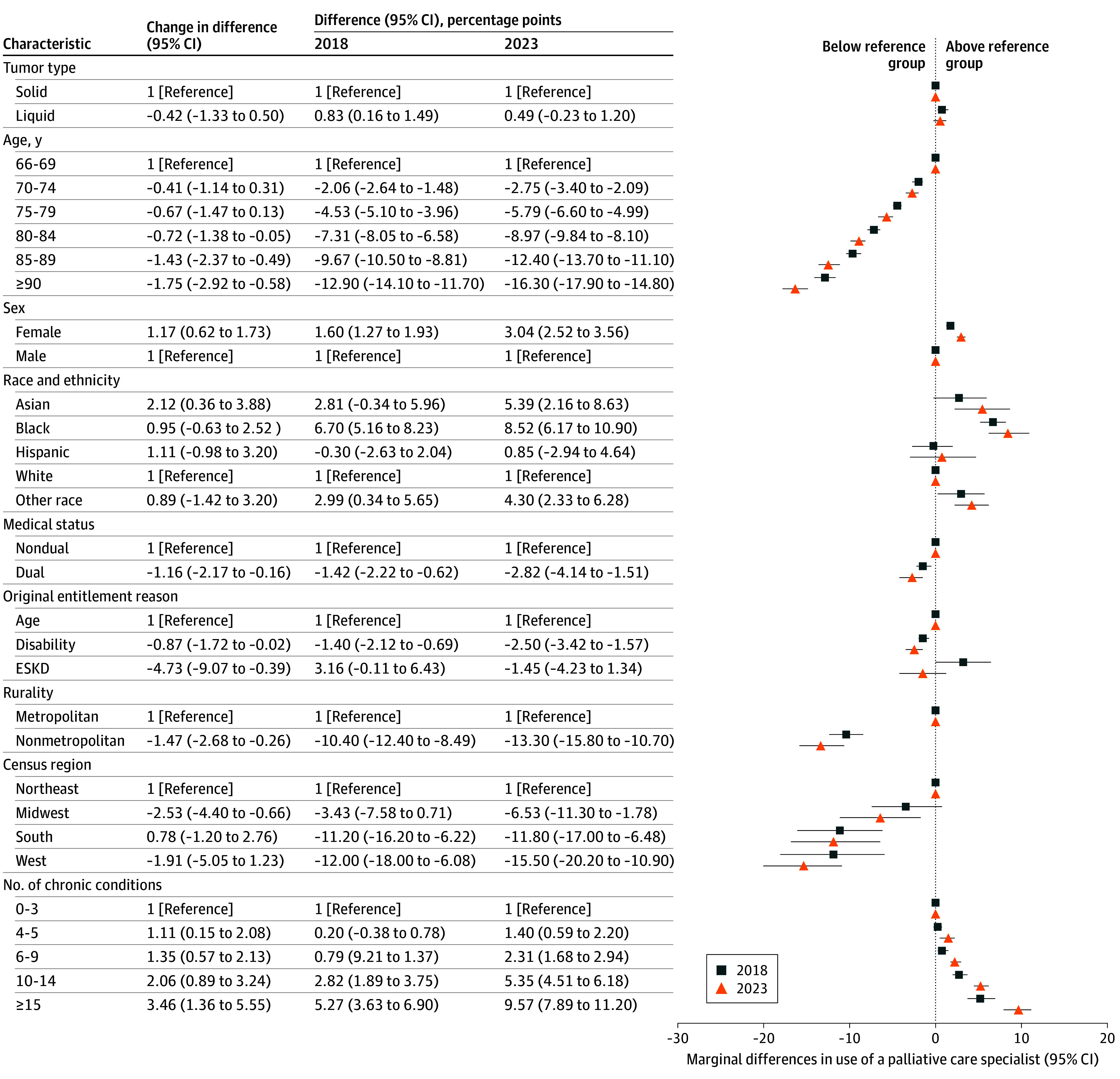
Changes in the Marginal Difference of Patient Characteristics for Any Use of Palliative Care Specialists The forest plot shows estimated marginal differences between patient characteristics in 2018 and 2023 in the percentage of decedents with any specialist use. Error bars indicate 95% CIs. Differences shown for each characteristic are in relation to their reference group. For example, nonmetropolitan decedents were 10.40 percentage points less likely to use a palliative care specialist than metropolitan decedents in 2018 and were 13.30 percentage points less likely in 2023. Between 2018 and 2023, nonmetropolitan decedents became 1.47 percentage points less likely than metropolitan decedents to use palliative care specialists. ESKD indicates end-stage kidney disease.

In our adjusted analysis (Figure 3), older age, lower income, living in a nonmetropolitan area, having disability or end-stage kidney disease as the original reason for entitlement (vs age), and living in the Midwest or West census regions were associated with less use of any PC specialists in 2018 and 2023. For example, in 2023, decedents 90 years or older were 16.30 (95% CI, 14.80-17.90) percentage points less likely to use any PC specialists compared with decedents aged 66 to 69 years; low-income decedents were 2.82 (95% CI, 1.51-4.14) percentage points less likely to use any PC specialists compared with non–low-income decedents; and decedents in nonmetropolitan areas were 13.30 (95% CI, 10.70-15.80) percentage points less likely to use any PC specialists compared with decedents in metropolitan areas.

Conversely, female sex, Asian race, and multiple chronic conditions were associated with more PC use in 2018 and 2023. In 2023, female decedents were 3.04 (95% CI, 2.52-3.56) percentage points more likely than male decedents to use any PC specialist; Asian decedents were 5.39 (95% CI, 2.16-8.63) percentage points more likely to use any PC specialist compared with White decedents; and decedents with at least 15 chronic conditions were 9.57 (95% CI, 7.89-11.20) percentage points more likely to use any PC specialist compared with decedents with 0 to 3 chronic conditions. For each subgroup, the marginal difference in PC specialist use in 2023 was consistent with 2018, if not slightly larger.

## Discussion

In this cohort study, there was a 24.2% increase in the fraction of decedents who had poor-prognosis cancers receiving any specialty PC encounters from 2018 to 2023, along with an 88.2% increase in patients receiving outpatient PC. Large shifts have occurred in how PC is delivered, with nearly one-fifth of these outpatient encounters delivered via telehealth, and by 2023, APCs were the most common clinician type delivering specialty PC. Despite these changes, differences in PC specialist use across patient characteristics largely remained unchanged during the same period, and only a minority had received specialty PC.^38,39^

Our findings are consistent with recent work that demonstrated that nurse practitioners constitute a significant proportion of the PC workforce^40^ and self-designated PC physicians are a shrinking proportion of clinicians delivering outpatient PC.^41^ Our study builds on this prior work by showing that APCs have supplanted PC physicians as the main clinicians providing outpatient PC. To our knowledge, this is the first study to broadly describe the rapid and continued growth of APCs in PC. The growing importance of APCs in PC is also consistent with larger trends in primary care and specialty care.^42^

Since the COVID-19 pandemic, telehealth use for PC has declined slightly but continued to play a sizeable role in outpatient PC, accounting for 18.2% of specialist encounters in 2023. While rates of telehealth use for PC were not as high as for psychiatry, telehealth use in PC was much higher than in oncology (<5%) and most other areas of clinical medicine.^43,44^ Telehealth may be well suited for PC treatment for patients with poor-prognosis cancers. Recent studies demonstrated telehealth’s equivalence to in-person PC in improving quality of life among patients with advanced lung cancer,^16^ and many patients with advanced cancer reported a preference for telehealth over in-person PC encounters due to less travel burden (which can be significant among patients with cancer^45,46^), increased convenience, and shorter wait times.^12^

Our study is also the first, to our knowledge, to demonstrate persistent differences in PC use across patient characteristics using nationally representative claims data. Despite increased use of PC specialists, as well as more variety in terms of how PC was being delivered (ie, more outpatient encounters, APC encounters, and telehealth use), differences in use across patient characteristics remained relatively stable between 2018 and 2023. One possibility is that APC growth is occurring in areas that already have physician specialists.^40^ Another is that increased overall adoption of telehealth may not have affected PC use in certain subgroups who were also less likely to adopt telehealth (eg, older patients, those with lower income, those living in nonmetropolitan areas).^47,48,49,50,51^ Therefore, mitigating differences in PC use may require targeting known preexisting PC access barriers^52^ and providing increased support for wider adoption of telehealth uptake.

### Limitations

There are several limitations to our study. First, the *ICD-10* code we used (Z51.5 for encounter with PC) to identify PC clinicians is not required for billing purposes when specialty PC is provided and therefore may lead to underidentification of PC specialists due to limited sensitivity.^28^ The code may be used for documenting services related to PC delivery (eg, transition to comfort care) that are not exclusive to PC specialists. To minimize these limitations, we identified clinicians as PC specialists in every year of our study period if they met our inclusion criteria in any year and set a high threshold (≥80%) for inclusion as a specialist by way of their clinical focus. Second, we only looked at E&M encounters delivered in the year prior to death. By doing so, we may have excluded PC services rendered earlier in the decedent’s illness trajectory. Third, our analysis is limited to E&M encounters that occurred before hospice enrollment and therefore does not reflect any hospice-related PC. To explore whether differences in PC across demographic groups may simply reflect differences in the timing of hospice enrollment, we estimated a sensitivity model of our subgroup analysis of PC use (Figure 3) that adjusted for days in hospice. The estimated differences in this model (eTable in Supplement 1) were consistent with our main results. Fourth, selecting a cohort conditioning on death may have introduced a selection bias due to variable survival times across cancer types. However, focusing on decedents who had poor-prognosis cancers mitigates some of this bias, since these patients are more likely to have shorter survival times and more predictable rates of decline. Finally, increased switching of Medicare beneficiaries from traditional fee-for-service to Medicare Advantage likely contributed to fewer decedents in 2023 compared with 2018.^53^ While evidence suggests differences in the composition of beneficiaries in traditional fee-for-service and Medicare Advantage plans,^54^ our cohort’s characteristics between 2018 and 2023 were similar (ie, standardized mean differences were all <0.1), making it less likely that the changes in outcomes were driven by compositional shifts in our sample.

## Conclusions

In this cohort study of decedents who had poor-prognosis cancers, there was substantial change in E&M encounters with PC specialists. PC delivery changed rapidly: APCs were the most common clinician type delivering specialty PC, and more decedents received outpatient PC, of which a sizeable proportion was delivered via telehealth and by APCs. However, differences in PC use for certain subgroups persisted, suggesting that different strategies may be needed to overcome barriers to PC access.

## Notes

Supplement 1. **eMethods 1.** Identifying Palliative Care Specialists in Claims **eMethods 2.** Poor-Prognosis Cancer Codes **eMethods 3.** Telehealth Codes **eTable.** Marginal Differences of Patient Characteristics on Any Use of Palliative Care Specialists in 2018 and 2023 With and Without Hospice Days Included as a Control **eFigure.** Flow Diagram of Study Cohort: Medicare Decedents With Poor-Prognosis Cancers, 2018 to 2023
eReferences.

Supplement 2.
Data Sharing Statement
