# Supplementary material for: Palliative Care Specialist Use Among Medicare Decedents Who Had Poor-Prognosis Cancers
Source: JAMA Netw Open. 2025 Jul 24;8(7):e2522886. doi: 10.1001/jamanetworkopen.2025.22886 (PMC12290731; doi:10.1001/jamanetworkopen.2025.22886)
Supplement: Supplement 1. — eMethods 1. Identifying Palliative Care Specialists in Claims eMethods 2. Poor-Prognosis Cancer Codes eMethods 3. Telehealth Codes eTable. Marginal Differences of Patient Characteristics on Any Use of Palliative Care Specialists in 2018 and 2023 With and Without Hospice Days Included as a Control eFigure. Flow Diagram of Study Cohort: Medicare Decedents With Poor-Prognosis Cancers, 2018 to 2023 eReferences. [file jamanetwopen-e2522886-s001.pdf]

Supplementary Online Content

Chua IS, Huskamp HA, Mehrotra A, Wilcock AD. Palliative care specialist use among Medicare decedents who had poor-prognosis cancers. *JAMA Netw Open.* 2025;8(7):e2522886. doi:10.1001/jamanetworkopen.2025.22886

eMethods 1. Identifying Palliative Care Specialists in Claims
eMethods 2. Poor-Prognosis Cancer Codes
eMethods 3. Telehealth Codes
eTable. Marginal Differences of Patient Characteristics on Any Use of Palliative Care Specialists in 2018 and 2023 With and Without Hospice Days Included as a Control
eFigure. Flow Diagram of Study Cohort: Medicare Decedents Who Had Poor-Prognosis Cancers, 2018 to 2023
eReferences.

This supplementary material has been provided by the authors to give readers additional information about their work.

© 2025 Chua IS et al. *JAMA Network Open*.

eMethods 1. Identifying Palliative Care Specialists in Claims

Overview

As described in the Methods, the focus in this study was on encounters with clinicians that specialize in palliative care (PC). We identified "PC specialists" as any clinician (identified as a unique National Provider Identification [NPI] number) that met at least one of the following two criteria:

(1) self-designation using Centers for Medicare and Medicaid Services (CMS) specialty code 17 for "Hospice and PC") in their Medicare enrollment application (which we referred to as "self-designated PC physicians"), or

(2) a clinician where at least 80% of their evaluation & management (E&M) encounters during a given year (among those with a minimum of 30 E&M encounters) had an International Classification of Disease Tenth Revision (ICD-10) code Z51.5 for "Encounter with PC."

Identification steps

To identify PC specialists, we used the Part B Carrier claims for all traditional Medicare beneficiaries over the period 2016 through 2023 (i.e., *not just for our cohort* but for all patients with Part B coverage, for any condition). E&M encounters were identified on the Carrier lines using the BETOS classification of the Healthcare Common Procedure Coding System (HCPCS) codes for "E&M Care." The following were our steps:

- We created clinician profiles by NPI, CMS specialty code and year, describing
    - their count of E&M claims, and
    - the share of E&M claims with a diagnosis code for PC.
- We removed clinician-years without ≥ 30 E&M claims.
- We kept clinician-years that met at least one of the following criteria:
    - the clinician's CMS specialty code was 17 indicating "Hospice and PC", and/or
    - ≥ 80% of their E&M claims had the ICD-10 code Z51.5 for "Encounter with PC."
- Using this set of clinician-years, we created 4 listings of NPIs:
    1. all PC specialists: the unique set of NPIs
    2. "self-designated" PC physician: the unique set of NPIs with CMS specialty code was 17
    3. advanced practice clinicians (APCs): the unique set of NPIs with CMS specialty code 50 for nurse practitioner or 97 for physician assistant
    4. "other physicians": the unique set of NPIs with any other CMS specialty code (not 17, 50 or 97)

If a clinician was identified as a "specialist" in 1 year, then we considered them a specialist in every year of our study. We chose this approach to minimize the possibility that we might be characterizing growth in using the Z51.5 diagnosis code as growth in the number of PC specialists over time. For example, suppose a nurse practitioner PC specialist started delivering care to patients in 2016 but was not using the Z51.5 diagnosis code on their claims until 2021. If we identified specialists within a given year, for example, then we would only call this clinician a PC specialist after they started using the code in 2021. Our approach was to call this clinician a specialist in "any year" we saw them delivering care to patients.

The impact of using the "any year" approach on the number of specialists we identified in our study is shown in the table below where we compare it to a "within year" approach, which identifies specialists as such only in the years they met our inclusion criteria.

	**"Any year"**			**"Within year"**		
	**2018**	**2023**	**% Chg**	**2018**	**2023**	**% Chg**
"Self-designated" physicians	1,737	1,955	13%	1,257	1,648	31%
APCs	2,876	4,201	46%	1,442	2,884	100%
Other physicians	1,119	1,160	4%	508	643	27%
Total	5,732	7,316	28%	3,207	5,175	61%

© 2025 Chua IS et al. *JAMA Network Open*.

The table shows the “any year” approach identifies many more specialists than the “within year” approach, with less growth between 2018 and 2023. This suggests that many specialists were delivering care to our study population before they started using the CMS code 17 or the PC diagnosis code Z51.5. While we cannot be 100% sure this was PC, we do know these were E&M encounters with poor prognosis cancer patients before (or after) a year in which the clinician was identified as a PC specialist. Moreover, the “any year” approach is less prone to overstating actual growth in specialists by way of greater use of the Z51.5 diagnosis code.

© 2025 Chua IS et al. *JAMA Network Open*.

eMethods 2. Poor-Prognosis Cancer Codes

Overview
Based on the prior work of Enzinger et al,[1,2] we created a list of ICD-10 codes of poor-prognosis cancers. This list combined the 10 most common causes of cancer death according to the American Cancer Society[3] and National Vital Statistics System.[4] We also included rare cancers that have a high mortality rate (e.g., cholangiocarcinoma). Additionally, certain solid tumors that tend to be diagnosed at earlier stages (e.g., breast, prostate, and colorectal) were required to have a concurrent non-lymphatic metastatic disease code to be considered a poor-prognosis cancer.

For future use of these codes, please cite Enzinger et al[1,2] in addition to our manuscript when referencing the poor-prognosis cancer cohort in the methods section.

Cancer Type	ICD-10 code	Metastatic required	Code description
Esophagus	C15	no	Malignant neoplasm of esophagus
	C153	no	Malignant neoplasm of upper third of esophagus
	C154	no	Malignant neoplasm of middle third of esophagus
	C155	no	Malignant neoplasm of lower third of esophagus. Excludes 1: -malignant neoplasm of cardio-esophageal junction
	C153	no	Malignant neoplasm of upper third of esophagus
	C154	no	Malignant neoplasm of middle third of esophagus
	C155	no	Malignant neoplasm of lower third of esophagus. Excludes 1: -malignant neoplasm of cardio-esophageal junction
	C158	no	Malignant neoplasm of overlapping sites of esophagus
	C159	no	Malignant neoplasm of esophagus, unspecified
Stomach	C16	no	Malignant neoplasm of stomach
	C160	no	Malignant neoplasm of cardia
	C164	no	Malignant neoplasm of pylorus
	C163	no	Malignant neoplasm of pyloric antrum
	C161	no	Malignant neoplasm of fundus of stomach
	C162	no	Malignant neoplasm of body of stomach
	C165	no	Malignant neoplasm of lesser curvature of stomach, unspecified
	C166	no	Malignant neoplasm of greater curvature of stomach, unspecified
	C168	no	Malignant neoplasm of overlapping sites of stomach
	C169	no	Malignant neoplasm of stomach, unspecified
Colon	C18	yes	Malignant neoplasm of colon. Excludes 1: -malignant carcinoid tumors of the colon (C7A.02-)
	C183	yes	Malignant neoplasm of hepatic flexure
	C184	yes	Malignant neoplasm of transverse colon
	C186	yes	Malignant neoplasm of descending colon
	C187	yes	Malignant neoplasm of sigmoid colon
	C180	yes	Malignant neoplasm of cecum
	C181	yes	Malignant neoplasm of appendix
	C182	yes	Malignant neoplasm of ascending colon
	C185	yes	Malignant neoplasm of splenic flexure
	C188	yes	Malignant neoplasm of overlapping sites of colon
	C189	yes	Malignant neoplasm of colon, unspecified
	C19	yes	Malignant neoplasm of rectosigmoid junction

© 2025 Chua IS et al. *JAMA Network Open*.

Cancer Type	ICD-10 code	Metastatic required	Code description
Rectum, Rectosigmoid junction, and Anus	C20	yes	Malignant neoplasm of rectum
	C211	yes	Malignant neoplasm of anal canal
	C210	yes	Malignant neoplasm of anus, unspecified
	C218	yes	Malignant neoplasm of overlapping sites of rectum, anus and anal canal
Malignant Neoplasms of Digestive Organs	C260	yes	Malignant neoplasm of intestinal tract, part unspecified
	C261	yes	Malignant neoplasm of spleen
	C269	yes	Malignant neoplasm of ill-defined sites within the digestive system
Liver	C22	no	Malignant neoplasm of liver and intrahepatic bile ducts
	C22.0	no	Liver cell carcinoma (approximate flag)
	C22.2	no	Hepatoblastoma (approximate flag)
	C22.7	no	Other specified carcinomas of liver (approximate flag)
	C22.8	no	Malignant neoplasm of liver, primary, unspecified as to type (approximate flag)
	C22.3	no	Angiosarcoma of liver. InclusionTerm: -Kupffer cell sarcoma (approximate flag)
	C22.4	no	Other sarcomas of liver (approximate flag)
	C22.1	no	Intrahepatic bile duct carcinoma
	C22.9	no	Malignant neoplasm of liver, not specified as primary or secondary
Gallbladder	C23	no	Malignant neoplasm of gallbladder
	C24	no	Malignant neoplasm of other and unspecified parts of biliary tract
	C24.0	no	Malignant neoplasm of extrahepatic bile duct
	C24.1	no	Malignant neoplasm of ampulla of Vater
	C24.8	no	Malignant neoplasm of overlapping sites of biliary tract
	C24.9	no	Malignant neoplasm of biliary tract, unspecified
Pancreas	C25	no	Malignant neoplasm of pancreas
	C250	no	Malignant neoplasm of head of pancreas
	C251	no	Malignant neoplasm of body of pancreas
	C252	no	Malignant neoplasm of tail of pancreas
	C253	no	Malignant neoplasm of pancreatic duct
	C254	no	Malignant neoplasm of endocrine pancreas
	C257	no	Malignant neoplasm of other parts of pancreas (Approximate Flag)
	C258	no	Malignant neoplasm of overlapping sites of pancreas (Approximate Flag)
	C259	no	Malignant neoplasm of pancreas, unspecified
Lung	C34	no	Malignant neoplasm of bronchus and lung
	C340	no	Malignant neoplasm of main bronchus
	C33	no	Malignant neoplasm of trachea
	C3400	no	Malignant neoplasm of unspecified main bronchus (Approximate Flag)
	C3401	no	Malignant neoplasm of main bronchus (Approximate Flag)
	C3402	no	Malignant neoplasm of main bronchus (Approximate Flag)
	C341	no	Malignant neoplasm of upper lobe, bronchus or lung
	C3410	no	Malignant neoplasm of upper lobe, unspecified bronchus or lung (Approximate Flag)

© 2025 Chua IS et al. *JAMA Network Open*.

Cancer Type	ICD-10 code	Metastatic required	Code description
	C3411	no	Malignant neoplasm of upper lobe, right bronchus or lung (Approximate Flag)
	C3412	no	Malignant neoplasm of upper lobe, left bronchus or lung (Approximate Flag)
	C342	no	Malignant neoplasm of middle lobe, bronchus or lung
	C343	no	Malignant neoplasm of lower lobe, bronchus or lung
	C3430	no	Malignant neoplasm of lower lobe, unspecified bronchus or lung (Approximate Flag)
	C3431	no	Malignant neoplasm of lower lobe, right bronchus or lung (approximate flag)
	C3432	no	Malignant neoplasm of lower lobe, left bronchus or lung
	C348	no	Malignant neoplasm of overlapping sites of bronchus and lung
	C3480	no	Malignant neoplasm of overlapping sites of unspecified bronchus and lung (Approximate Flag)
	C3481	no	Malignant neoplasm of overlapping sites of right bronchus and lung (Approximate Flag)
	C3482	no	Malignant neoplasm of overlapping sites of left bronchus and lung (Approximate Flag)
	C349	no	Malignant neoplasm of unspecified part of bronchus or lung
	C3490	no	Malignant neoplasm of unspecified part of unspecified bronchus or lung (Approximate Flag)
	C3491	no	Malignant neoplasm of unspecified part of right bronchus or lung (Approximate Flag)
	C3492	no	Malignant neoplasm of unspecified part of left bronchus or lung (Approximate Flag)
	C39	no	Malignant neoplasm of other and ill-defined sites in the respiratory system and intrathoracic organs
	C390	no	Malignant neoplasm of upper respiratory tract, part unspecified
	C399	no	Malignant neoplasm of lower respiratory tract, part unspecified
Secondary Neoplasms	C780	N/A	Secondary malignant neoplasm of lung
	C7800	N/A	Secondary malignant neoplasm of unspecified lung
	C7801	N/A	Secondary malignant neoplasm of right lung
	C7802	N/A	Secondary malignant neoplasm of left lung
	C781	N/A	Secondary malignant neoplasm of mediastinum
	C782	N/A	Secondary malignant neoplasm of pleura
	C783	N/A	Secondary malignant neoplasm of other and unspecified respiratory organs
	C7830	N/A	Secondary malignant neoplasm of unspecified respiratory organ
	C7839	N/A	Secondary malignant neoplasm of other respiratory organs
	C784	N/A	Secondary malignant neoplasm of small intestine
	C785	N/A	Secondary malignant neoplasm of large intestine and rectum
	C786	N/A	Secondary malignant neoplasm of retroperitoneum and peritoneum
	C787	N/A	Secondary malignant neoplasm of liver and intrahepatic bile duct

© 2025 Chua IS et al. *JAMA Network Open*.

Cancer Type	ICD-10 code	Metastatic required	Code description
	C788	N/A	Secondary malignant neoplasm of other and unspecified digestive organs
	C7880	N/A	Secondary malignant neoplasm of unspecified digestive organ
	C7889	N/A	Secondary malignant neoplasm of other digestive organs
	C79	N/A	Secondary malignant neoplasm of other and unspecified sites
	C790	N/A	Secondary malignant neoplasm of kidney and renal pelvis
	C7900	N/A	Secondary malignant neoplasm of unspecified kidney and renal pelvis
	C7901	N/A	Secondary malignant neoplasm of right kidney and renal pelvis
	C791	N/A	Secondary malignant neoplasm of bladder and other and unspecified urinary organs
	C7910	N/A	Secondary malignant neoplasm of unspecified urinary organs
	C7911	N/A	Secondary malignant neoplasm of bladder
	C7919	N/A	Secondary malignant neoplasm of other urinary organs
	C792	N/A	Secondary malignant neoplasm of skin
	C793	N/A	Secondary malignant neoplasm of brain and cerebral meninges
	C7931	N/A	Secondary malignant neoplasm of brain
	C7932	N/A	Secondary malignant neoplasm of cerebral meninges
	C7940	N/A	Secondary malignant neoplasm of unspecified part of nervous system
	C7949	N/A	Secondary malignant neoplasm of other parts of nervous system
	C795	N/A	Secondary malignant neoplasm of bone and bone marrow
	C7951	N/A	Secondary malignant neoplasm of bone
	C7952	N/A	Secondary malignant neoplasm of bone marrow
	C796	N/A	Secondary malignant neoplasm of ovary
	C7960	N/A	Secondary malignant neoplasm of unspecified ovary
	C7961	N/A	Secondary malignant neoplasm of right ovary
	C7962	N/A	Secondary malignant neoplasm of left ovary
	C797	N/A	Secondary malignant neoplasm of adrenal gland
	C7970	N/A	Secondary malignant neoplasm of unspecified adrenal gland
	C7971	N/A	Secondary malignant neoplasm of right adrenal gland
	C7972	N/A	Secondary malignant neoplasm of left adrenal gland
	C798	N/A	Secondary malignant neoplasm of other specified sites
	C7981	N/A	Secondary malignant neoplasm of breast
	C7982	N/A	Secondary malignant neoplasm of genital organs
	C7989	N/A	Secondary malignant neoplasm of other specified sites
	C799	N/A	Secondary malignant neoplasm of unspecified site
Melanoma	C43	yes	Malignant melanoma of skin
	C430	yes	Malignant melanoma of lip (Approximate Flag)
	D030	yes	Melanoma in situ of lip (Approximate Flag)
	C431	yes	Malignant melanoma of eyelid, including canthus
	C4310	yes	Malignant melanoma of unspecified eyelid, including canthus (Approximate Flag)

© 2025 Chua IS et al. *JAMA Network Open*.

Cancer Type	ICD-10 code	Metastatic required	Code description
	D0310	yes	Melanoma in situ of unspecified eyelid, including canthus (Approximate Flag)
	D0311	yes	Melanoma in situ of right eyelid, including canthus (Approximate Flag)
	D0312	yes	Melanoma in situ of left eyelid, including canthus (Approximate Flag)
	C4311	yes	Malignant melanoma of right eyelid, including canthus
	C4312	yes	Malignant melanoma of left eyelid, including canthus
	C432	yes	Malignant melanoma of ear and external auricular canal
	C4320	yes	Malignant melanoma of unspecified ear and external auricular canal (Approximate Flag)
	D0320	yes	Melanoma in situ of unspecified ear and external auricular canal (Approximate Flag)
	D0321	yes	Melanoma in situ of right ear and external auricular canal (Approximate Flag)
	D0322	yes	Melanoma in situ of left ear and external auricular canal (Approximate Flag)
	C4321	yes	Malignant melanoma of right ear and external auricular canal
	C4322	yes	Malignant melanoma of left ear and external auricular canal
	C433	yes	Malignant melanoma of other and unspecified parts of face
	C4330	yes	Malignant melanoma of unspecified part of face (Approximate Flag)
	C4331	yes	Malignant melanoma of nose (Approximate Flag)
	C4339	yes	Malignant melanoma of other parts of face (Approximate Flag)
	D0330	yes	Melanoma in situ of unspecified part of face (Approximate Flag)
	D0339	yes	Melanoma in situ of other parts of face (Approximate Flag)
	C434	yes	Malignant melanoma of scalp and neck (Approximate Flag)
	D034	yes	Melanoma in situ of scalp and neck (Approximate Flag)
	C435	yes	Malignant melanoma of trunk
	C4359	yes	Malignant melanoma of other part of trunk (Approximate Flag)
	D0351	yes	Melanoma in situ of anal skin (Approximate Flag)
	D0352	yes	Melanoma in situ of breast (skin) (soft tissue) (Approximate Flag)
	D0359	yes	Melanoma in situ of other part of trunk (Approximate Flag)
	C4352	yes	Malignant melanoma of skin of breast
	C4351	yes	Malignant melanoma of anal skin
	C436	yes	Malignant melanoma of upper limb, including shoulder
	C4360	yes	Malignant melanoma of unspecified upper limb, including shoulder (Approximate Flag)
	D0360	yes	Melanoma in situ of unspecified upper limb, including shoulder (Approximate Flag)
	D0361	yes	Melanoma in situ of right upper limb, including shoulder (Approximate Flag)

© 2025 Chua IS et al. *JAMA Network Open*.

Cancer Type	ICD-10 code	Metastatic required	Code description
	D0362	yes	Melanoma in situ of left upper limb, including shoulder (Approximate Flag)
	C4361	yes	Malignant melanoma of right upper limb, including shoulder
	C4362	yes	Malignant melanoma of left upper limb, including shoulder
	C437	yes	Malignant melanoma of lower limb, including hip
	C4370	yes	Malignant melanoma of unspecified lower limb, including hip (Approximate Flag)
	D0370	yes	Melanoma in situ of unspecified lower limb, including hip (Approximate Flag)
	D0371	yes	Melanoma in situ of right lower limb, including hip (Approximate Flag)
	D0372	yes	Melanoma in situ of left lower limb, including hip (Approximate Flag)
	C4371	yes	Malignant melanoma of right lower limb, including hip
	C4372	yes	Malignant melanoma of left lower limb, including hip
	C438	yes	Malignant melanoma of overlapping sites of skin (Approximate Flag)
	D038	yes	Melanoma in situ of other sites (Approximate Flag)
	C439	yes	Malignant melanoma of skin, unspecified (Approximate Flag)
	D039	yes	Melanoma in situ, unspecified (Approximate Flag)
Breast	C50	yes	Malignant neoplasm of breast
	C50019	yes	Malignant neoplasm of nipple and areola, unspecified female breast
	C50011	yes	Malignant neoplasm of nipple and areola, right female breast
	C50012	yes	Malignant neoplasm of nipple and areola, left female breast
	C50119	yes	Malignant neoplasm of central portion of unspecified female breast
	C50111	yes	Malignant neoplasm of central portion of right female breast
	C50112	yes	Malignant neoplasm of central portion of left female breast
	C501	yes	Malignant neoplasm of central portion of breast
	C5011	yes	Malignant neoplasm of central portion of breast, female
	C5001	yes	Malignant neoplasm of nipple and areola, female
	C502	yes	Malignant neoplasm of upper-inner quadrant of breast
	C5021	yes	Malignant neoplasm of upper-inner quadrant of breast, female
	C50219	yes	Malignant neoplasm of upper-inner quadrant of unspecified female breast
	C50211	yes	Malignant neoplasm of upper-inner quadrant of right female breast
	C50212	yes	Malignant neoplasm of upper-inner quadrant of left female breast
	C503	yes	Malignant neoplasm of lower-inner quadrant of breast
	C5031	yes	Malignant neoplasm of lower-inner quadrant of breast, female

© 2025 Chua IS et al. *JAMA Network Open*.

Cancer Type	ICD-10 code	Metastatic required	Code description
	C50319	yes	Malignant neoplasm of lower-inner quadrant of unspecified female breast
	C50311	yes	Malignant neoplasm of lower-inner quadrant of right female breast
	C50312	yes	Malignant neoplasm of lower-inner quadrant of left female breast
	C54	yes	Malignant neoplasm of corpus uteri
	C504	yes	Malignant neoplasm of upper-outer quadrant of breast
	C5041	yes	Malignant neoplasm of upper-outer quadrant of breast, female
	C50419	yes	Malignant neoplasm of upper-outer quadrant of unspecified female breast
	C50411	yes	Malignant neoplasm of upper-outer quadrant of right female breast
	C50412	yes	Malignant neoplasm of upper-outer quadrant of left female breast
	C505	yes	Malignant neoplasm of lower-outer quadrant of breast
	C5051	yes	Malignant neoplasm of lower-outer quadrant of breast, female
	C50519	yes	Malignant neoplasm of lower-outer quadrant of unspecified female breast
	C50511	yes	Malignant neoplasm of lower-outer quadrant of right female breast
	C50512	yes	Malignant neoplasm of lower-outer quadrant of left female breast
	C506	yes	Malignant neoplasm of axillary tail of breast
	C5061	yes	Malignant neoplasm of axillary tail of breast, female
	C50619	yes	Malignant neoplasm of axillary tail of unspecified female breast
	C50611	yes	Malignant neoplasm of axillary tail of right female breast
	C50612	yes	Malignant neoplasm of axillary tail of left female breast
	C508	yes	Malignant neoplasm of overlapping sites of breast
	C5081	yes	Malignant neoplasm of overlapping sites of breast, female
	C50819	yes	Malignant neoplasm of overlapping sites of unspecified female breast
	C50811	yes	Malignant neoplasm of overlapping sites of right female breast
	C50812	yes	Malignant neoplasm of overlapping sites of left female breast
	C509	yes	Malignant neoplasm of breast of unspecified site
	C50919	yes	Malignant neoplasm of unspecified site of unspecified female breast
	C50911	yes	Malignant neoplasm of unspecified site of right female breast
	C50912	yes	Malignant neoplasm of unspecified site of left female breast
	C50021	yes	Malignant neoplasm of nipple and areola, right male breast
	C50022	yes	Malignant neoplasm of nipple and areola, left male breast
	C50029	yes	Malignant neoplasm of nipple and areola, unspecified male breast

© 2025 Chua IS et al. *JAMA Network Open*.

Cancer Type	ICD-10 code	Metastatic required	Code description
	C50121	yes	Malignant neoplasm of central portion of right male breast
	C50122	yes	Malignant neoplasm of central portion of left male breast
	C50129	yes	Malignant neoplasm of central portion of unspecified male breast
	C50221	yes	Malignant neoplasm of upper-inner quadrant of right male breast
	C50222	yes	Malignant neoplasm of upper-inner quadrant of left male breast
	C50229	yes	Malignant neoplasm of upper-inner quadrant of unspecified male breast
	C50321	yes	Malignant neoplasm of lower-inner quadrant of right male breast
	C50322	yes	Malignant neoplasm of lower-inner quadrant of left male breast
	C50329	yes	Malignant neoplasm of lower-inner quadrant of unspecified male breast
	C50421	yes	Malignant neoplasm of upper-outer quadrant of right male breast
	C50422	yes	Malignant neoplasm of upper-outer quadrant of left male breast
	C50429	yes	Malignant neoplasm of upper-outer quadrant of unspecified male breast
	C50521	yes	Malignant neoplasm of lower-outer quadrant of right male breast
	C50522	yes	Malignant neoplasm of lower-outer quadrant of left male breast
	C50529	yes	Malignant neoplasm of lower-outer quadrant of unspecified male breast
	C50621	yes	Malignant neoplasm of axillary tail of right male breast
	C50622	yes	Malignant neoplasm of axillary tail of left male breast
	C50629	yes	Malignant neoplasm of axillary tail of unspecified male breast
	C50821	yes	Malignant neoplasm of overlapping sites of right male breast
	C50822	yes	Malignant neoplasm of overlapping sites of left male breast
	C50829	yes	Malignant neoplasm of overlapping sites of unspecified male breast
	C50922	yes	Malignant neoplasm of unspecified site of left male breast
	C50921	yes	Malignant neoplasm of unspecified site of right male breast
	C50929	yes	Malignant neoplasm of unspecified site of unspecified male breast
	C5091	yes	Malignant neoplasm of breast of unspecified site, female
Ovary	C56	yes	Malignant neoplasm of ovary
	C569	yes	Malignant neoplasm of unspecified ovary
	C561	yes	Malignant neoplasm of right ovary
	C562	yes	Malignant neoplasm of left ovary
	C5700	yes	Malignant neoplasm of unspecified fallopian tube
	C5701	yes	Malignant neoplasm of right fallopian tube

© 2025 Chua IS et al. *JAMA Network Open*.

Cancer Type	ICD-10 code	Metastatic required	Code description
	C5702	yes	Malignant neoplasm of left fallopian tube
	C5710	yes	Malignant neoplasm of unspecified broad ligament
	C5711	yes	Malignant neoplasm of right broad ligament
	C5712	yes	Malignant neoplasm of left broad ligament
	C573	yes	Malignant neoplasm of parametrium
	C5720	yes	Malignant neoplasm of unspecified round ligament
	C5721	yes	Malignant neoplasm of right round ligament
	C5722	yes	Malignant neoplasm of left round ligament
	C574	yes	Malignant neoplasm of uterine adnexa, unspecified
	C577	yes	Malignant neoplasm of other specified female genital organs
	C578	yes	Malignant neoplasm of overlapping sites of female genital organs
	C574	yes	Malignant neoplasm of uterine adnexa, unspecified
	C579	yes	Malignant neoplasm of female genital organ, unspecified
	C57	yes	Malignant neoplasm of other and unspecified female genital organs
	C570	yes	Malignant neoplasm of fallopian tube
	C571	yes	Malignant neoplasm of broad ligament
	C572	yes	Malignant neoplasm of round ligament
Prostate	C61	yes	Malignant neoplasm of prostate
Bladder	C67	no	Malignant neoplasm of bladder
	C670	no	Malignant neoplasm of trigone of bladder
	C671	no	Malignant neoplasm of dome of bladder
	C672	no	Malignant neoplasm of lateral wall of bladder
	C68	no	Malignant neoplasm of other and unspecified urinary organs
	C673	no	Malignant neoplasm of anterior wall of bladder
	C680	no	Malignant neoplasm of urethra
	C674	no	Malignant neoplasm of posterior wall of bladder
	C681	no	Malignant neoplasm of paraurethral glands
	C675	no	Malignant neoplasm of bladder neck
	C676	no	Malignant neoplasm of ureteric orifice
	C677	no	Malignant neoplasm of urachus
	C678	no	Malignant neoplasm of overlapping sites of bladder
	C679	no	Malignant neoplasm of bladder, unspecified
	C688	no	Malignant neoplasm of overlapping sites of urinary organs
	C689	no	Malignant neoplasm of urinary organ, unspecified
Kidney	C64	yes	Malignant neoplasm of kidney, except renal pelvis
	C649	yes	Malignant neoplasm of unspecified kidney, except renal pelvis
	C641	yes	Malignant neoplasm of right kidney, except renal pelvis
	C642	yes	Malignant neoplasm of left kidney, except renal pelvis
	C65	yes	Malignant neoplasm of renal pelvis
	C659	yes	Malignant neoplasm of unspecified renal pelvis
	C651	yes	Malignant neoplasm of right renal pelvis
	C652	yes	Malignant neoplasm of left renal pelvis
	C669	yes	Malignant neoplasm of unspecified ureter
	C680	yes	Malignant neoplasm of urethra
	C681	yes	Malignant neoplasm of paraurethral glands

© 2025 Chua IS et al. *JAMA Network Open*.

Cancer Type	ICD-10 code	Metastatic required	Code description
	C688	yes	Malignant neoplasm of overlapping sites of urinary organs
	C689	yes	Malignant neoplasm of urinary organ, unspecified
Brain	C710	no	Malignant neoplasm of cerebrum, except lobes and ventricles
	C711	no	Malignant neoplasm of frontal lobe
	C712	no	Malignant neoplasm of temporal lobe
	C713	no	Malignant neoplasm of parietal lobe
	C714	no	Malignant neoplasm of occipital lobe
	C715	no	Malignant neoplasm of cerebral ventricle
	C716	no	Malignant neoplasm of cerebellum
	C717	no	Malignant neoplasm of brain stem
	C718	no	Malignant neoplasm of overlapping sites of brain
	C719	no	Malignant neoplasm of brain, unspecified
Brain Uncertain	D432	no	Neoplasm of uncertain behavior of brain, unspecified
	D434	no	Neoplasm of uncertain behavior of spinal cord
Spinal	C709	no	Malignant neoplasm of meninges, unspecified
	C700	no	Malignant neoplasm of cerebral meninges
	C701	no	Malignant neoplasm of spinal meninges
	C79.32	no	Secondary malignant neoplasm of cerebral meninges
	C79.49	no	Secondary malignant neoplasm of other parts of nervous system
Spinal 2	C72	no	Malignant neoplasm of spinal cord, cranial nerves and other parts of central nervous system
	C720	no	Malignant neoplasm of spinal cord
	C721	no	Malignant neoplasm of cauda equina
	C7220	no	Malignant neoplasm of unspecified olfactory nerve
	C7221	no	Malignant neoplasm of right olfactory nerve
	C7222	no	Malignant neoplasm of left olfactory nerve
	C7230	no	Malignant neoplasm of unspecified optic nerve
	C7231	no	Malignant neoplasm of right optic nerve
	C7232	no	Malignant neoplasm of left optic nerve
	C7240	no	Malignant neoplasm of unspecified acoustic nerve
	C7241	no	Malignant neoplasm of right acoustic nerve
	C7242	no	Malignant neoplasm of left acoustic nerve
	C7250	no	Malignant neoplasm of unspecified cranial nerve
	C7259	no	Malignant neoplasm of other cranial nerves
	C725	no	Malignant neoplasm of other and unspecified cranial nerves
	C729	no	Malignant neoplasm of central nervous system, unspecified
Carcinoma Unknown	C80	no	Malignant neoplasm without specification of site
	C800	no	Disseminated malignant neoplasm, unspecified
	C801	no	Malignant (primary) neoplasm, unspecified
	C802	no	Malignant neoplasm associated with transplanted organ
Leukemia	C91	no	Lymphoid leukemia
	C910	no	Acute lymphoblastic leukemia [ALL]
	C9100	no	Acute lymphoblastic leukemia not having achieved remission
	C9101	no	Acute lymphoblastic leukemia, in remission
	C9102	no	Acute lymphoblastic leukemia, in relapse
	C92	no	Myeloid leukemia

© 2025 Chua IS et al. *JAMA Network Open*.

Cancer Type	ICD-10 code	Metastatic required	Code description
	C920	no	Acute myeloblastic leukemia
	C925	no	Acute myelomonocytic leukemia
	C926	no	Acute myeloid leukemia with 11q23-abnormality
	C92A	no	Acute myeloid leukemia with multilineage dysplasia
	C9200	no	Acute myeloblastic leukemia, not having achieved remission
	C9240	no	Acute promyelocytic leukemia, not having achieved remission
	C9250	no	Acute myelomonocytic leukemia, not having achieved remission
	C9260	no	Acute myeloid leukemia with 11q23-abnormality not having achieved remission
	C92A0	no	Acute myeloid leukemia with multilineage dysplasia, not having achieved remission
	C9201	no	Acute myeloblastic leukemia, in remission
	C9241	no	Acute promyelocytic leukemia, in remission
	C9251	no	Acute myelomonocytic leukemia, in remission
	C9261	no	Acute myeloid leukemia with 11q23-abnormality in remission
	C92A1	no	Acute myeloid leukemia with multilineage dysplasia, in remission
	C9202	no	Acute myeloblastic leukemia, in relapse
	C9242	no	Acute promyelocytic leukemia, in relapse
	C9252	no	Acute myelomonocytic leukemia, in relapse
	C9262	no	Acute myeloid leukemia with 11q23-abnormality in relapse
	C92A2	no	Acute myeloid leukemia with multilineage dysplasia, in relapse
	C95	no	Leukemia of unspecified cell type
	C950	no	Acute leukemia of unspecified cell type
	C9500	no	Acute leukemia of unspecified cell type not having achieved remission
	C9110	no	Chronic lymphocytic leukemia of B-cell type not having achieved remission
	C9111	no	Chronic lymphocytic leukemia of B-cell type in remission
	C9112	no	Chronic lymphocytic leukemia of B-cell type in relapse
	C91A	no	Mature B-cell leukemia Burkitt-type
	C91Z	no	Other lymphoid leukemia
	C9130	no	Prolymphocytic leukemia of B-cell type not having achieved remission
	C9150	no	Adult T-cell lymphoma/leukemia (HTLV-1-associated) not having achieved remission
	C9160	no	Prolymphocytic leukemia of T-cell type not having achieved remission
	C91A0	no	Mature B-cell leukemia Burkitt-type not having achieved remission
	C91Z0	no	Other lymphoid leukemia not having achieved remission
	C9131	no	Prolymphocytic leukemia of B-cell type, in remission
	C9151	no	Adult T-cell lymphoma/leukemia (HTLV-1-associated), in remission
	C9161	no	Prolymphocytic leukemia of T-cell type, in remission

© 2025 Chua IS et al. *JAMA Network Open*.

Cancer Type	ICD-10 code	Metastatic required	Code description
	C91A1	no	Mature B-cell leukemia Burkitt-type, in remission
	C91Z1	no	Other lymphoid leukemia, in remission
	C9132	no	Prolymphocytic leukemia of B-cell type, in relapse
	C9152	no	Adult T-cell lymphoma/leukemia (HTLV-1-associated), in relapse
	C9162	no	Prolymphocytic leukemia of T-cell type, in relapse
	C91A2	no	Mature B-cell leukemia Burkitt-type, in relapse
	C91Z2	no	Other lymphoid leukemia, in relapse
	C9140	no	Hairy cell leukemia not having achieved remission
	C9141	no	Hairy cell leukemia, in remission
	C9142	no	Hairy cell leukemia, in relapse
	C919	no	Lymphoid leukemia, unspecified
	C9190	no	Lymphoid leukemia, unspecified not having achieved remission
	C9191	no	Lymphoid leukemia, unspecified, in remission
	C9192	no	Lymphoid leukemia, unspecified, in relapse
	C9210	no	Chronic myeloid leukemia, BCR/ABL-positive, not having achieved remission
	C9211	no	Chronic myeloid leukemia, BCR/ABL-positive, in remission
	C9212	no	Chronic myeloid leukemia, BCR/ABL-positive, in relapse
	C9220	no	Atypical chronic myeloid leukemia, BCR/ABL-negative, not having achieved remission
	C9221	no	Atypical chronic myeloid leukemia, BCR/ABL-negative, in remission
	C9222	no	Atypical chronic myeloid leukemia, BCR/ABL-negative, in relapse
	C9230	no	Myeloid sarcoma, not having achieved remission
	C9231	no	Myeloid sarcoma, in remission
	C9232	no	Myeloid sarcoma, in relapse
	C929	no	Myeloid leukemia, unspecified
	C9290	no	Myeloid leukemia, unspecified, not having achieved remission
	C9291	no	Myeloid leukemia, unspecified in remission
	C9292	no	Myeloid leukemia, unspecified in relapse
	C92Z	no	Other myeloid leukemia
	C92Z0	no	Other myeloid leukemia not having achieved remission
	C92Z1	no	Other myeloid leukemia, in remission
	C92Z2	no	Other myeloid leukemia, in relapse
	C93	no	Monocytic leukemia
	C930	no	Acute monoblastic/monocytic leukemia
	C9300	no	Acute monoblastic/monocytic leukemia, not having achieved remission
	C9301	no	Acute monoblastic/monocytic leukemia, in remission
	C9302	no	Acute monoblastic/monocytic leukemia, in relapse
	C9310	no	Chronic myelomonocytic leukemia not having achieved remission
	C9311	no	Chronic myelomonocytic leukemia, in remission
	C9312	no	Chronic myelomonocytic leukemia, in relapse
	C9330	no	Juvenile myelomonocytic leukemia, not having achieved remission

© 2025 Chua IS et al. *JAMA Network Open*.

Cancer Type	ICD-10 code	Metastatic required	Code description
	C93Z0	no	Other monocytic leukemia, not having achieved remission
	C9331	no	Juvenile myelomonocytic leukemia, in remission
	C93Z1	no	Other monocytic leukemia, in remission
	C9332	no	Juvenile myelomonocytic leukemia, in relapse
	C93Z2	no	Other monocytic leukemia, in relapse
	C9390	no	Monocytic leukemia, unspecified, not having achieved remission
	C9391	no	Monocytic leukemia, unspecified in remission
	C9392	no	Monocytic leukemia, unspecified in relpase
	C9501	no	Acute leukemia of unspecified cell type, in remission
	C9502	no	Acute leukemia of unspecified cell type, in relapse
	C9510	no	Chronic leukemia of unspecified cell type not having achieved remission
	C9511	no	Chronic leukemia of unspecified cell type, in remission
	C9512	no	Chronic leukemia of unspecified cell type, in relapse
	C959	no	Leukemia, unspecified
	C9590	no	Leukemia, unspecified not having achieved remission
	C9591	no	Leukemia, unspecified, in remission
	C9592	no	Leukemia, unspecified, in relapse

© 2025 Chua IS et al. *JAMA Network Open*.

eMethods 3. Telehealth Codes

Overview

We identified telehealth utilization using place of service codes and/or HCPCS codes and modifiers for audio-only or video telehealth encounters. We considered the encounter a telehealth encounter if it included any of the following criteria:

1. Place of service codes 02, 10
2. Modifier codes GT, GQ, 95, G0, FQ, FR, 93
3. HCPCS codes
    - G0425-7 (telehealth consultations emergency department or initial inpatient)
    - G2025 (when a FQHC / Rural health clinic provides telemedicine services)
    - G0406-8 (follow-up inpatient or skilled nursing facility consultations via telehealth)
    - G0459 (pharmacologic management service furnished via telehealth to inpatients)
    - G0508-9 (telehealth consultations for critical care)
    - 0188T - Remote real-time interactive video-conferenced critical care, first 30- 74 minutes.
    - 99441–99443
    - 98966–98968
    - G0320: Home health services furnished using synchronous telemedicine - video
    - G0321: Home health services furnished using synchronous telemedicine - audio-only
    - G0322: The collection of physiologic data via RPM for home health
    - C7900-C7902: Partial Hospitalization and Intensive Outpatient Program services to take place in a patient's home via telehealth,

Audio-only telehealth	99441–99443, 98966–98968, G0321, modifiers FQ or 93
Video telehealth	any telemedicine visits that is not categorized above

© 2025 Chua IS et al. *JAMA Network Open*.

eTable. Marginal Differences of Patient Characteristics on Any Use of Palliative Care Specialists in 2018 and 2023 With and Without Hospice Days Included as a Control

	**Marginal Differences in 2018 (95% CI)**		**Marginal Differences in 2023 (95% CI)**		**Change in marginal differences (95% CI)**	
	Without hospice days	With hospice days	Without hospice days	With hospice days	Without hospice days	With hospice days
Days in hospice	NA	-0.04 (-0.05, -0.04)	NA	-0.05 (-0.05, -0.04)	NA	0 (-0.01, 0.00)
Liquid tumor	0.83 (0.16, 1.49)	0.31 (-0.36, 0.99)	0.485 (-0.23, 1.20)	-0.01 (-0.74, 0.72)	-0.42 (-1.33, 0.50)	-0.34 (-1.26, 0.57)
70-74	-2.06 (-2.64, -1.48)	-2.04 (-2.63, -1.45)	-2.75 (-3.40, -2.09)	-2.66 (-3.31, -2.02)	-0.41 (-1.14, 0.31)	-0.35 (-1.08, 0.38)
75-79	-4.53 (-5.10, -3.96)	-4.42 (-5.00, -3.85)	-5.79 (-6.60, -4.99)	-5.6 (-6.39, -4.81)	-0.67 (-1.47, 0.13)	-0.60 (-1.39, 0.20)
80-84	-7.31 (-8.05, -6.58)	-7.01 (-7.75, -6.28)	-8.97 (-9.84, -8.10)	-8.59 (-9.47, -7.71)	-0.72 (-1.38, -0.05)	-0.67 (-1.34, 0.01)
85-89	-9.67 (-10.50, -8.81)	-9.13 (-9.97, -8.29)	-12.4 (-13.70, -11.10)	-11.7 (-13.00, -10.40)	-1.43 (-2.37, -0.49)	-1.36 (-2.31, -0.40)
90+	-12.9 (-14.10, -11.70)	-12.1 (-13.2, -10.90)	-16.3 (-17.90, -14.80)	-15.2 (-16.80, -13.50)	-1.75 (-2.92, -0.58)	-1.51 (-2.73, -0.30)
Female	1.60 (1.27, 1.93)	1.90 (1.55, 2.24)	3.04 (2.52, 3.56)	3.41 (2.88, 3.93)	1.17 (0.62, 1.73)	1.21 (0.64, 1.78)
Asian	2.81 (-0.34, 5.96)	2.55 (-0.58, 5.68)	5.39 (2.16, 8.63)	5.15 (1.89, 8.41)	2.12 (0.36, 3.88)	2.16 (0.42, 3.91)
Black	6.70 (5.16, 8.23)	6.54 (5.00, 8.09)	8.52 (6.17, 10.90)	8.26 (5.90, 10.60)	0.95 (-0.63, 2.52)	0.86 (-0.70, 2.41)
Hispanic	-0.30 (-2.63, 2.04)	-0.41 (-2.78, 1.96)	0.85 (-2.94, 4.64)	0.71 (-3.08, 4.49)	1.11 (-0.97, 3.20)	1.11 (-1.01, 3.22)
Other race	2.99 (0.344, 5.65)	2.92 (0.29, 5.54)	4.30 (2.33, 6.28)	4.19 (2.23, 6.15)	0.89 (-1.42, 3.20)	0.86 (-1.44, 3.17)
Dual	-1.42 (-2.22, -0.62)	-1.03 (-1.83, -0.23)	-2.82 (-4.14, -1.51)	-2.25 (-3.57, -0.94)	-1.16 (-2.17, -0.16)	-1.04 (-2.04, -0.04)
Disability	-1.40 (-2.12, -0.69)	-1.26 (-1.98, -0.54)	-2.50 (-3.42, -1.57)	-2.29 (-3.21, -1.37)	-0.87 (-1.72, -0.02)	-0.83 (-1.67, 0.01)
ESKD	3.16 (-0.11, 6.43)	2.58 (-0.66, 5.83)	-1.45 (-4.23, 1.34)	-2.13 (-4.91, 0.65)	-4.73 (-9.07, -0.39)	-4.76 (-9.04, -0.48)
Non-Metro	-10.40 (-12.40, -8.49)	-10.50 (-12.50, -8.51)	-13.3 (-15.80, -10.70)	-13.30 (-15.90, -10.80)	-1.47 (-2.68, -0.26)	-1.45 (-2.66, -0.24)
Midwest	-3.43 (-7.58, 0.712)	-3.16 (-7.33, 1.00)	-6.53 (-11.30, -1.78)	-6.18 (-10.90, -1.41)	-2.53 (-4.40, -0.66)	-2.48 (-4.37, -0.59)
South	-11.20 (-16.20, -6.22)	-10.90 (-15.90, -5.90)	-11.80 (-17.00, -6.48)	-11.30 (-16.60, -6.07)	0.78 (-1.20, 2.76)	0.87 (-1.13, 2.87)
West	-12.00 (-18.00, -6.08)	-11.80 (-17.80, -5.74)	-15.50 (-20.20, -10.90)	-15.20 (-19.80, -10.50)	-1.91 (-5.05, 1.23)	-1.81 (-5.00, 1.38)
4-5 conditions	0.20 (-0.38, 0.78)	0.32 (-0.26, 0.90)	1.40 (0.59, 2.20)	1.55 (0.76, 2.34)	1.11 (0.15, 2.08)	1.13 (0.16, 2.09)
6-9 conditions	0.79 (0.21, 1.37)	1.03 (0.46, 1.61)	2.31 (1.68, 2.94)	2.61 (1.99, 3.23)	1.35 (0.57, 2.13)	1.37 (0.58, 2.16)
10-14 conditions	2.82 (1.89, 3.75)	3.17 (2.23, 4.11)	5.35 (4.51, 6.18)	5.83 (4.99, 6.66)	2.06 (0.89, 3.24)	2.14 (0.96, 3.33)
15+ conditions	5.27 (3.63, 6.90)	5.75 (4.10, 7.41)	9.57 (7.89, 11.2)	10.2 (8.46, 11.90)	3.46 (1.36, 5.55)	3.50 (1.35, 5.64)

© 2025 Chua IS et al. *JAMA Network Open*.

eFigure. Flow Diagram of Study Cohort: Medicare Decedents Who Had Poor-Prognosis Cancers, 2018 to 2023

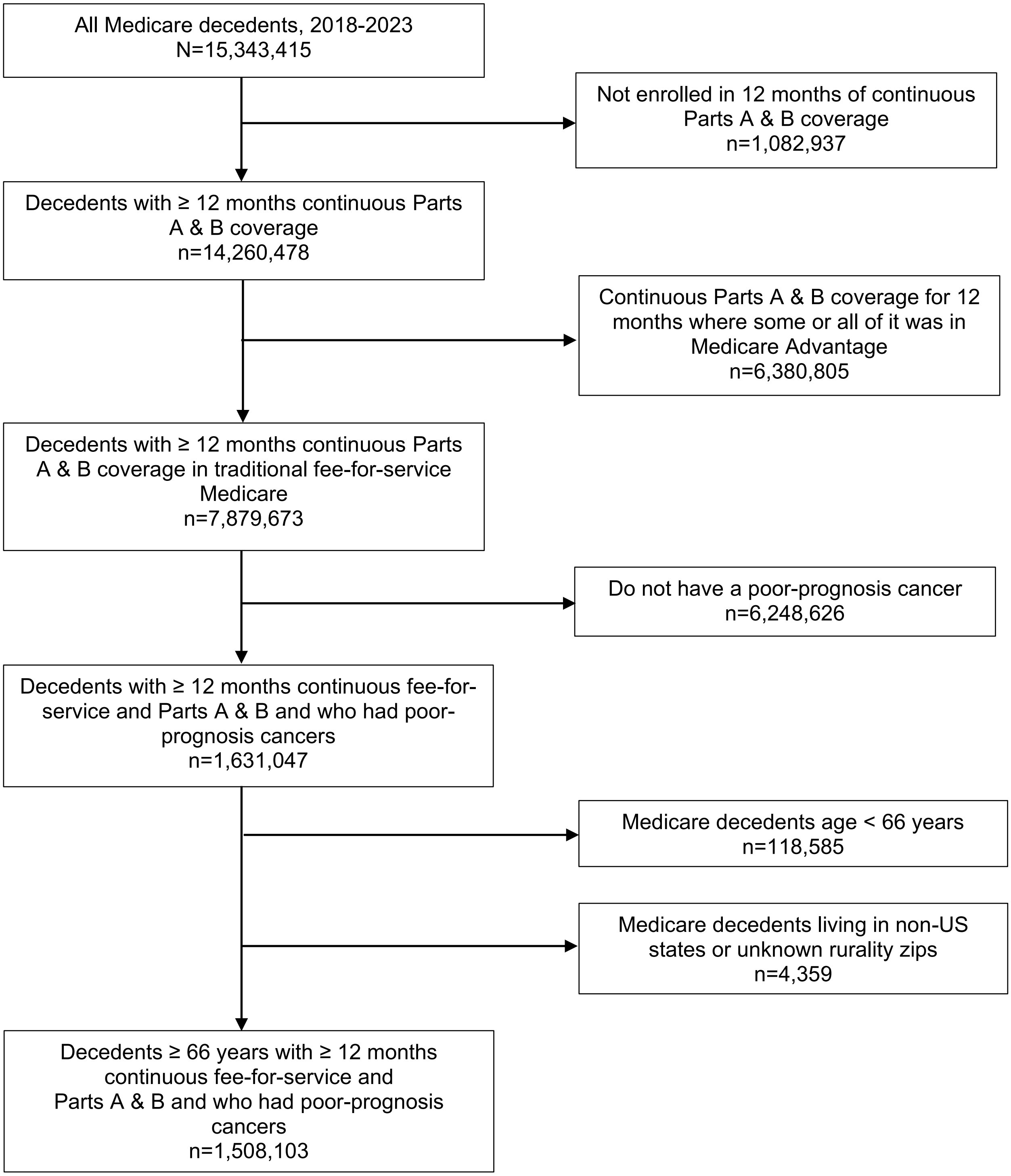

© 2025 Chua IS et al. *JAMA Network Open*.

eReferences.

1. Enzinger AC, Ghosh K, Keating NL, Cutler DM, Landrum MB, Wright AA. US Trends in Opioid Access Among Patients With Poor Prognosis Cancer Near the End-of-Life. *J Clin Oncol.* 2021;39(26):2948-2958.
2. Enzinger AC, Ghosh K, Keating NL, et al. Racial and Ethnic Disparities in Opioid Access and Urine Drug Screening Among Older Patients With Poor-Prognosis Cancer Near the End of Life. *J Clin Oncol.* 2023;41(14):2511-2522.
3. Siegel RL, Giaquinto AN, Jemal A. Cancer statistics, 2024. *CA: A Cancer Journal for Clinicians.* 2024;74(1):12-49.
4. Centers for Disease Control and Prevention: National Vital Statistics System. https://www.cdc.gov/nchs/nvss/index.htm?CDC_AA_refVal=https%3A%2F%2Fwww.cdc.gov%2Fnchs%2Fnvss.htm. Accessed July 17, 2024.

© 2025 Chua IS et al. *JAMA Network Open*.
