# Supplementary material for: Palliative Care Specialist Use Among Medicare Decedents Who Had Poor-Prognosis Cancers
Source: JAMA Netw Open. 2025 Jul 24;8(7):e2522886. doi: 10.1001/jamanetworkopen.2025.22886 (PMC12290731; doi:10.1001/jamanetworkopen.2025.22886)
Supplement: Supplement 2. — Data Sharing Statement [file jamanetwopen-e2522886-s002.pdf]

Data Sharing Statement

Chua. Changes in Palliative Care Specialist Use Among Medicare Decedents Who Had Poor-Prognosis Cancers. *JAMA Netw Open*. Published July 24, 2025.
doi:10.1001/jamanetworkopen.2025.22886

Data
Data available: No
